# Supplementary material for: The Evolution and Characterization of the RNA Interference Pathways in Lophotrochozoa
Source: Genome Biol Evol. 2024 May 7;16(5):evae098. doi: 10.1093/gbe/evae098 (PMC11114477; doi:10.1093/gbe/evae098)
Supplement: evae098_Supplementary_Data [file evae098_supplementary_data.zip › Supplementary_materials.zip!/selected_miRNAs/NoGe_selected_miRNAs/known_miRNA_families_pdf_files/NMRB01000015.1_Notospermus_geniculatus_strain_Ushimado_scaffold15,_whole_genome_shotgun_sequence_640.pdf]

Provisional ID	:	NMRB01000015.1_Notospermus_geniculatus_strain_Ushimado_scaffold15,_whole_genome_shotgun_sequence_640
Score total	:	3690891.4
Score for star read(s)	:	3.9
Score for read counts	:	3690882.7
Score for mfe	:	1.8
Score for randfold	:	0
Score for cons. seed	:	3
Total read count	:	7239519
Mature read count	:	7239351
Loop read count	:	0
Star read count	:	168

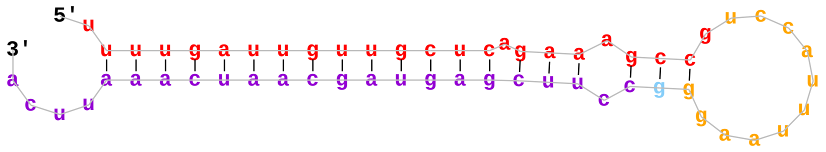

Genome coordinates: NMRB01000015.1 491747-491806

pre-miRNA sequence:
UUUUGAUUGUUGCUCAGAAAGCCGUUUUGAUUGUUGCUCAGAAAGCCGUCCAUUUAAGGGCCUUCGAGUAGCAAUCAAAUUCA

Mature sequence: UUUUGAUUGUUGCUCAGAAAGCCG

Aligned mature sequence of the *N. geniculatus* pre-miRNA with the top 5 hits from the BLAST search against the pre-miRNA mirGeneDB set:

```
                           ***********************
Nge_Mir-315                uuuugauuguugcucagaaagccg
Bge-Mir-315_5p             uuuugauuguugcucagaaagcc-
Lgi-Mir-315_5p             uuuugauuguugcucagaaagcc-
Dma-Mir-315_5p             uuuugauuguugcucagaaagcc-
Dpu-Mir-315_5p             uuuugauuguugcucagaaagcc-
Aae-Mir-315_5p             uuuugauuguugcucagaaagcc-
```
